# Impact of Frozen Storage on Sensory, Physicochemical, and Volatile Compounds Parameters of Different Extra Virgin Olive Oils

**DOI:** 10.3390/foods13233764

**Published:** 2024-11-24

**Authors:** Enrique J. Díaz-Montaña, María Barbero-López, Ramón Aparicio-Ruiz, Diego L. García-González, María T. Morales

**Affiliations:** 1 Department of Analytical Chemistry, Faculty of Pharmacy, University of Seville, Prof. García González, 2, 41012 Seville, Spain; edmontana@us.es (E.J.D.-M.); aparicioruiz@us.es (R.A.-R.); 2 Instituto de la Grasa (CSIC), Edificio 46, Ctra. de Utrera, Km. 1, 41013 Sevilla, Spain; mbarbero@ig.csic.es (M.B.-L.); dlgarcia@ig.csic.es (D.L.G.-G.)

**Keywords:** oil samples, sensory profile, secondary oxidation, volatile compounds, rancidity

## Abstract

Storage is important for virgin olive oil, a product obtained only during the harvest period, which requires a year-round storage until its best-before date. Low temperatures slow undesirable reactions, though this method is not widely applied. The objective of this paper is to assess the impact of frozen storage on the volatile composition and sensory properties of virgin olive oils. The quality parameters, volatile composition, and sensory profiles were analyzed for samples stored under different conditions (time 0, sixth month supermarket and frozen storage, and long-term-frozen). The physicochemical parameters of the samples stored under supermarket conditions showed significant differences (*p* < 0.05), with the frozen-storage sample after three months of storage. Additionally, the samples stored under supermarket conditions showed higher volatile concentrations than frozen ones, with increased concentrations of aldehydes and acids producing sensory defects. Thirty-two samples, considered as the long-term-frozen, were divided into three groups depending on the frozen-storage time (1, 6 or 10 years). These long-term-frozen storage samples confirmed the suitability of the proposed oxidation markers (pentanal, hexanal, heptanal, nonanal, acetic, propanoic, butanoic, and hexanoic acids) for differentiating storage conditions and times. This work highlights the oxidation process under different storage conditions and suggests oxidation markers.

## 1. Introduction

High-quality virgin olive oil, considered by the current regulations [1], is obtained when sound and good-quality olives are used and properly processed. The extra-virgin olive oil category is the most appreciated due to the pleasant aroma and the absence of sensory defects [2]. The volatiles, aroma-related compounds, of these outstanding virgin olive oils can come from different routes, such as fatty acid metabolism, amino-acid metabolism, and homolytic cleavage of 13-hydroperoxides, but they mainly come from the lipoxygenase (LOX) pathway. The LOX route consists of an enzymatic oxidation that leads to the formation of C6 volatile compounds, such as *(E)*-2-hexenal or *(Z)*-3-hexenol and their esters, which are responsible for the green and fruity sensory attributes of virgin olive oil [3]. The proportion of C5 and C6 volatile compounds depends on several factors, such as the geographical origin, harvesting, irrigation, and variety. The volatile composition can be used as a fingerprint to differentiate virgin olive oils [4,5]. Moreover, when an extra-virgin olive oil is rich in C5 and C6 volatile compounds and has an exceptionally high sensory quality, it is currently labeled as premium, although this category is not considered in the current regulations [6].

Although the whole process of the virgin olive oil production is carried out under optimal conditions, it begins to oxidize from the moment it is obtained, first enzymatically through the LOX route, producing pleasant-aroma-related volatile compounds, and then chemically, producing unpleasant, rancid-related compounds, among others [7]. Chemical oxidation processes include auto-oxidation, photo-oxidation, and thermo-oxidation. Thermo-oxidation involves complex variables and typically produces hydroperoxides from alkyl and peroxyl radicals. Photo-oxidation is significant due to photosensitizers like chlorophyll acting as pro-oxidants under light, even though in darkness, other compounds, like phenols, can act as anti-oxidants, slowing down the oxidative process. Auto-oxidation, the primary oxidative process in lipid foods, occurs spontaneously through a free-radical chain mechanism [8,9,10,11,12,13]. Oxidation of unsaturated fatty acids (UFAs) leads to hydroperoxides that alter the volatile composition, replacing initial C5 and C6 volatiles with oxidation-related compounds like aldehydes and acids [7,14]. The main UFAs in virgin olive oil are oleic, linoleic, and linolenic acids. Oxidation of oleic acid forms octanal, nonanal, 2-decenal, and decanal; linoleic acid oxidation produces pentanal, hexanal, heptanal, 2-octenal, 2-nonenal, and 2,4-decadienal; linolenic acid oxidation produces propanal, butanal, 2-butenal, 2-pentenal, 2-hexenal, 3,6-nonadienal, and decatrienal [15]. Prolonged oxidation further converts aldehydes into carboxylic acids. These oxidation-derived aldehydes and acids are responsible for the rancid defect and are proposed as markers of the oxidative process [16,17].

The oxidation process in virgin olive oil leads to quality loss, impacting its classification and causing economic losses [18]. Since olive oil is produced only during the harvest season (mainly three months), it needs to be stored for most of the year, making storage conditions crucial for maintaining quality [19].

Numerous studies have examined the effects of light, temperature, and the package headspace gas on olive oil during storage, typically under conditions used by producers or supermarkets [20,21]. High temperature effects are well-studied, but low temperature effects are less so. Refrigeration (4 °C) and freezing (−20 °C) slow down undesirable reactions, such as lipid auto-oxidation and the formation of volatile compounds affecting aroma. However, low-temperature storage is not commonly used for olive oil, except for preserving samples for analysis [22,23]. Some studies indicate low-temperature storage prevents the quality decay of virgin olive oils [24,25,26]. However, contradictory findings exist regarding the oxidative stability of thawed oils due to potential crystallization and precipitation of hydrophilic compounds, like phenols. Some authors suggest that, due to the crystallization of phenols, the oxidative stability of thawed oils might change, because not the whole phenolic fraction will be reintegrated in the oil matrix [27,28]; meanwhile, other authors showed that there are no big differences between the solid and liquid phase when olive oils are stored at refrigeration temperatures [29].

There are not too many studies focused on freezing as a preservation option, and they are mainly focused on the physicochemical parameters, phenolic composition, and LOX-derived compounds [6,27,30,31,32]. Therefore, the aim of this work was to study the generation of secondary oxidation products in a control sample, to establish volatile-oxidation markers applicable to samples of different varieties and harvesting periods, and to assess the impact of long-term frozen storage on volatile compounds and sensory properties of a broad number of samples, with particular emphasis on the rancid sensory defect.

## 2. Materials and Methods

### 2.1. Chemicals

The reagents employed were of pure analytical grade. Acetic acid (99%), *(Z)*-3-hexenyl acetate (98%), pentan-3-ol (98%), *(Z)*-2-hexen-1-ol (96%), hexanoic acid (99%), heptanoic acid (99%), nonanoic acid (96%), *(Z)*-3-hexenal (95%), *(E)*-2-hexen-1-ol (96%), *(Z)*-3-hexen-1-ol (98%), and hexyl acetate (99%), were purchased from Merck (Darmstadt, Germany). Isobutyl acetate (99%), used as internal standard, was also purchased from Merck (Darmstadt, Germany).

Heptanal (95%), 6-methyl-5-hepten-2-one (99%), and 3-pentanone (99.5%) were purchased from Panreac (Barcelona, Spain).

Propanoic acid (99%), butanoic acid (99%), pentanoic acid (99%), *(E)*-3-heptenal (98%), hexanal (98%), hexanol (98%), nonanal (95%), octanal (98%), pentanal (98%), 1-penten-3-one (98%), and *(E)*-2-hexenal (97%) were purchased from Fluka (Zwijndrecht, The Netherlands).

### 2.2. Samples and Storage Conditions

Extra-virgin olive oil (var. Manzanilla; 2021/2022 harvesting) purchased from a local market (Sevilla, Andalucía, Spain) was used as control sample to study the oxidation process and to select the oxidation markers under different storage conditions. The control sample (VOO-0) was analyzed, and its quality category established according to the current regulation [1]. Then, the VOO-0 sample was divided into two aliquots; one of them was stored in six one-liter bottles for six months under supermarket storage conditions (22 ± 2 °C; 400–600 lx) with cycle of 12 h light/darkness and labeled as commercially stored virgin olive oil (CVOO). The other aliquot was stored also in six one-liter bottles at −20 °C in darkness and labeled as frozen virgin olive oil (FVOO). Thus, CVOO and FVOO samples came from the control sample (VOO-0) but were subjected to two different storage conditions. Once per month, the quality parameters, volatile composition, and sensory profile were determined on CVOO and FVOO samples.

In addition, 32 samples were used for a long-term freezing-storage experiment. These samples were originally of the extra-virgin olive oil category and were purchased from local markets (Sevilla, Andalucía, Spain) (Table 1). They were repackaged in 1 L dark glass bottles and frozen at −20 °C in darkness. These 32 extra-virgin olive oil samples were divided in three batches depending on the storage time (Table 1). The first batch (L1.1.–L1.10.) was made up of 10 samples from 2021/2022 harvesting, and they were stored for 12 months (1 year). The second batch (L2.1.–L2.10.) was composed of 10 samples from the 2016/2017 campaign, and they were stored for 72 months (6 years). The third batch (L3.1.–L3.12.) were 12 samples from the 2012/2013 harvest, which were stored for 120 months (10 years). As shown in Table 1, most of them were from different Spanish regions, and only a few were from other Mediterranean countries. Samples were from different cultivars, Picual, Hojiblanca, and Arbequina being the most common ones. The wide variability of the samples allowed for obtaining general information about the oxidative process and drawing general conclusions of oxidation under freezing-storage conditions not biased by cultivar or geographical origin.

After the storage period, all the samples were thawed for two hours at room temperature (22 ± 2 °C) in the absence of light before the analyses.

### 2.3. Quality Parameters

The physicochemical parameters analyzed were in concordance with the EU regulation in force at the moment of the samples acquisition [1,33,34,35] and were determined in triplicate for free acidity (FA), peroxide value (PV), and specific extinction values (K_232_, K_270_). All the reagents used in this study were of pure analytical grade purchased from Merck (Darmstadt, DE, Germany). The spectrophotometer used was a UV-3100PC from VWR Avantor (Philadelphia, PA, USA).

### 2.4. Sensory Analysis

The samples were subjected to the sensory analysis before freezing them. The method used was that established by the European regulations in force at the time of acquisition [1,33,34,35].

In addition, after the storage period, once the samples were thawed, a sensory evaluation was carried out by a quantitative–descriptive analysis (QDA) using a specific protocol developed for this study. The protocol includes an evaluation sheet that was created considering the quantification of sensory attributes intensity. An unstructured 5 cm scale (Figure S1) was used to evaluate the attributes (green, fruity, and tomato) and defects (rancid, fusty/muddy sediment, greasy, winey/vinegary, and musty/humid). In both cases, positive attributes and sensory defects, two free options, labeled as “other”, were considered; thus, the panelist could add extra information they considered important. The sensory evaluation was performed by eight assessors.

### 2.5. Volatile Compounds Determination (DHS-GC-FID)

The volatile compounds analysis was performed by placing 1.5 g of the sample and 150 μL of internal standard (isobutyl acetate) into a 20 mL glass vial. After capping the vial with a silicone/polytetrafluoroethylene (PTFE) septum, the sample was preheated for 18 min at 40 °C and mixed for 15 min. Thereafter, a flowrate of 5 mL/min of helium was used to move the volatiles from the headspace of the vial to a Tenax TATM trap. This procedure was carried out using a HT3 Dynamic Headspace System (DHS) (Teledyne Tekmar, Mason, OH, USA).

The volatile desorption was carried out at 260 °C for 5 min in a 7:1 split mode, onto a TR-WAX capillary column (60 m × 0.25 mm i.d., 0.25 μm; Teknokroma, Spain) in a Varian 3900 gas chromatograph (GC) (Palo Alto, CA, USA) with a flame ionization detector (FID), using hydrogen with a flowrate of 1.5 mL/min as carrier gas. The oven was programmed in two steps: (i) isotherm 10 min at 35 °C; (ii) an increase of 3 °C/min until 200 °C, which was held for 1 min. The FID temperature was settled at 280 °C, the signal being recorded with a Star Chromatography Workstation, System Control version 6 (Palo Alto, CA, USA).

The determination of volatile compounds was carried out in triplicate. The identification of the volatiles was carried out using pure analytical grade standards and the quantification using the internal standard methodological calibration.

### 2.6. Statistical Analysis

The data were transferred from the Star Chromatography Workstation to Excel 2016 (Microsoft Corp., Redmond, WA, USA). Excel was used to perform the univariate analysis (outlier detection, calculation of means, standard deviations, etc.) and as an interface between the data obtained by the chromatographic software and the statistical software Statistica version 8.0 (Statsoft; Tulsa, OK, USA), which was used to perform the multivariate analysis of the data. Student’s t-test was employed to detect significant differences (*p* < 0.05) between group means. A principal component analysis (PCA) was carried out using the proposed volatile-oxidation markers as variables to assess the relationship between the sample types.

## 3. Results and Discussion

### 3.1. CVOO and FVOO Samples: Storage Effect and Oxidation Markers Selection

The values of the quality parameters of the control sample (VOO-0) allowed its classification as extra-virgin olive oil: acidity index = 0.45%, peroxide value = 4.00 meq O_2_/kg, K_232_ = 1.50, K_270_ = 0.19, ∆K = 0.0018, and fatty acid ethyl esters = 8.0 mg/kg. As described above, this sample was divided into CVOO (stored at 22 °C) and FVOO (stored at −20 °C). Over time, all quality parameters of the CVOO sample increased significantly (*p* < 0.05), with K_270_ exceeding regulation limits [1] by the third month and the acidity index by the sixth month. K_270_ indicates the presence of secondary oxidation products, while increased free acidity results from triglyceride hydrolysis. Consequently, CVOO changed from extra-virgin to virgin olive oil from the third month onwards, highlighting the quality loss due to oxidation during storage. Although a VOO usually remains in the same category for a longer time, sometimes, some factors, such as the balance of anti-oxidants and pro-oxidants, in combination to light, may lead to a faster change in category. Conversely, the FVOO sample parameters remained within regulation limits [1] and showed a non-significant increase (*p* < 0.05). Thus, FVOO retained its extra-virgin quality, likely due to the lower rate of oxidation process at low temperatures.

Table 2 shows whether there are significant differences (*p* < 0.05) in the main quality parameters between the CVOO and FVOO samples, thus reflecting the effect of storage conditions on the oxidative process. During the first two months, due to the onset of the oxidative process, the differences were not significant (*p* < 0.05). However, from the third month, when the CVOO sample changed category, significant differences (*p* < 0.05) were observed between both samples (FVOO and CVOO). These differences were not only observed in the K_270_ parameter overstepped in the CVOO sample but also in the acidity index. After five months of storage, both samples showed significant differences in all the parameters, except for the peroxide value, which contrasts with what was reported by [31], who observed differences in all the parameters. It was in the sixth month when significant differences (*p* < 0.05) were observed in all the parameters, probably due to the higher deterioration of the CVOO sample (stored at 22 °C) in comparison to the FVOO sample (stored at −20 °C).

As well as the quality parameters, the volatile composition of the control sample (VOO-0) changed differently between the CVOO and FVOO samples over time. The volatile profile of VOO-0 sample was rich in C5 and C6 compounds, especially carbonyl compounds, alcohols, and their esters, which are responsible for the green and fruity sensory attributes of virgin olive oil [2]. Additionally, several compounds related to sensory defects, such as heptanal or acetic acid, were quantified in the control sample at a low concentration. However, after six months of storage, more oxidation-derived compounds, such as nonanal or hexanoic acid, were identified and quantified in the CVOO and FVOO samples.

In the CVOO sample, those compounds derived from the LOX pathway related to the green-fruity sensory perception, such as hexyl acetate or *(E)*-2-hexenal, decreased significantly (*p* < 0.05) during the storage, which is in agreement with other authors [2,18,36]. However, the concentration of the compounds derived from oxidation increased. Pentanal and hexanal, which originally contribute to green-fruity sensory perceptions, when at low concentrations, are generated due to the chemical oxidation of linoleic acid reaching higher concentrations and contributing to rancid off flavor, so they are considered oxidation-derived compounds [4,15]. The pentanal concentration increased significantly (*p* < 0.05) during the storage; whereas hexanal concentration was maintained because it was generated by the oxidative process while being oxidized to hexanoic acid. These results are in concordance with other authors [17,37]. In addition, the concentration of those compounds that are only produced by the autooxidation process, such as nonanal or hexanoic acid, increased significantly (*p* < 0.05) during the six-month storage, except heptanal. Nonanal is an oxidation product of the oleic acid and, during the storage, presented significant (*p* < 0.05) differences between almost all months. In contrast to the control sample (VOO-0) where nonanal was not detected, the CVOO sample stored for six months showed a concentration of 2.67 ± 0.13 mg/kg. After six months of storage, the CVOO sample also showed a significant increase (*p* < 0.05) of acetic, propanoic, butanoic, and hexanoic acids, reaching a concentration of 4.04 ± 0.28, 1.89 ± 0.22, 6.15 ± 0.31, and 7.63 ± 0.37 mg/kg, respectively.

On the other hand, in the FVOO sample, the green-related compounds, such as hexyl acetate or *(E)*-2-hexenal, did not show downward trends, and they maintained their concentration with a mean variation of 7.1%, which agrees with other authors [6,31]. Although these compounds are not markers of autooxidation, as they are produced by other pathways, they should be initially considered, as their concentration values vary as the oxidative process progresses. Currently, the oxidation-related compounds, such as nonanal, are commonly used as markers, but they are not normally studied in frozen samples. These oxidation compounds showed a significant increase (*p* < 0.05) as in the CVOO sample, except for hexanal and heptanal, which only increased during the frozen storage. Hexanal increased its concentration due to the slowdown of the oxidative process, which ensured that it did not degrade to hexanoic acid. In the case of heptanal, which had a concentration of 1.04 ± 0.20 mg/kg in the control sample (VOO-0), it reached a concentration of 1.79 ± 0.23 mg/kg. Heptanal is not considered to be produced during storage in dark conditions [21], which is in contrast with the results observed in the FVOO sample. Thus, it should be interesting to consider heptanal as marker of the storage in absence of light, studying its capacity to differentiate the samples.

**Figure 1:**
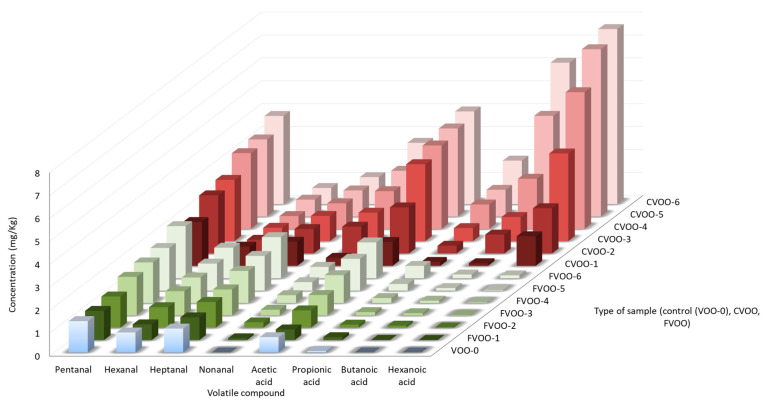
Concentration (mg/kg) of the proposed volatile-oxidation markers in the VOO-0 (blue), FVOO (green), and CVOO (red) samples. Note: next to the sample type is the storage-time in month.

Figure 1 displays the concentration of the volatile compounds that showed the greatest differences between CVOO and FVOO samples and that are proposed to be oxidation markers at different storage conditions. As can be seen, the concentration of oxidation-related volatile compounds increased in the CVOO sample more than in the FVOO sample, highlighting the extent of the protective effect of frozen storage on the volatile fraction. The aldehydes showed two different trends over time, the first one observed in pentanal and nonanal, and the second one detected in hexanal and heptanal. Pentanal and nonanal presented an increasing trend in both samples, the generation rate being higher in the CVOO sample; whereas hexanal and heptanal concentration did not vary in the CVOO sample and increased in the FVOO sample. The four acids proposed as markers showed the same trend, presenting a significant increase (*p* < 0.05) in both groups of samples (CVOO and FVOO). However, there is a significant difference (*p* < 0.05) between the acid generation rate in CVOO samples in comparison to FVOO samples. Due to the slowdown of the oxidative process at −20 °C, the production of acids is much lower, presenting between a 61.3 and a 98.4% less concentration in the FVOO sample. In general, the total volatile concentration in the CVOO sample, after three months, was significantly higher (*p* < 0.05) in comparison to the concentration of the FVOO sample at any moment of the storage (except for hexanal and heptanal). It is remarkable that three months of storage was also the time needed to observe significant differences (*p* < 0.05) in the quality parameters. The protective role of dark conditions and low temperatures was also proved after six-month storage, where the CVOO sample doubled the concentration of most oxidation-related volatile compounds in comparison to the FVOO sample.

When storage is carried out under accelerated conditions, a similar behavior of volatiles is observed but in hours (between 50 and 70 h) [2]. Accelerated storage is considered when medium–high temperatures (40–100 °C) and oxygen flow, or oxygen in the headspace, are applied to accelerate the oxidative process. Under these conditions, extra-virgin olive oil can degrade and generate aldehydes and acids, passing to lower quality categories such as virgin or lampante olive oil. In the case of LOX derivatives, such as *(Z)*-3-hexenal or hexyl acetate, they eventually disappear [2,38]. While other compounds, such as nonanal, increase due to the oxidation of unsaturated fatty acids [39,40]. Therefore, the variation in the volatiles concentration in samples stored under room-temperature and accelerated conditions is similar [2,41]. In contrast to the storage carried out at low temperatures, it has been observed that quality parameters are less affected by the oxidative process, and that volatiles derived from LOX are not displaced by the oxidation-derived volatiles, preserving olive oil quality.

Besides the determination of the quality parameters and the volatile compounds, the changes in the sensory profile, to relate the appearance of the rancid defect with the chemical changes, were also evaluated. In the control sample (VOO-0), the main descriptors were those related to green/fruity sensory perception (Table 3). In the case of the CVOO sample, an incipient rancidity began to be detected from the third month onwards, and positive attributes such as apple were no longer appreciated, losing the extra-virgin category (Table 3).

The detection of sensory defects supports what is observed in the quality parameters and volatile profile, where aldehydes and acids increased over time. Thus, in the CVOO sample, the decrease in green-related compounds, due to the increase in oxidation-related compounds, was responsible for the changes observed in the sensory profile. On the contrary, the FVOO sample (stored at −20 °C) maintained the same sensory profile as the control sample (VOO-0) because, during the storage, the LOX-derived compounds were not displaced by the oxidation products due to the slowing down of the oxidative process.

Thus, volatile compounds coming from the LOX pathway, responsible for the green/fruity sensory perceptions, were not used as markers because they did not vary their concentration in short frozen storage (6 months). On the other hand, the volatile compounds coming from chemical oxidation, which are mainly responsible for unpleasant aromas [3] and were selected as oxidation markers were those shown in Figure 1: pentanal, hexanal, heptanal, nonanal, acetic acid, propanoic acid, butanoic acid, and hexanoic acid. The selected markers matched with the selection of volatiles in other studies in which the degradation of quality in virgin olive oils are also studied [21,42].

Therefore, the oxidative process that a sample can undergoes is highly influenced by the storage conditions. The conditions of the CVOO sample (22 ± 2 °C; 400–600 lx; cycle of 12 h light/darkness) led to a lower quality, producing a change from extra-virgin to virgin olive oil category [1] from the third month onwards. This category change can be observed from multiple perspectives including quality parameters, volatile composition, and sensory profile. On the contrary, FVOO sample, stored at −20 °C in dark conditions, did not present a category change, even though oxidation-related compounds slightly increased their concentrations. Thus, the study of CVOO and FVOO samples from different perspectives allowed for the selection of general and suitable oxidation volatile markers.

Once the oxidation of the CVOO and FVOO samples were studied and the volatile-oxidation markers were selected, the next step was to analyze the oxidation process that a set of highly diverse samples (different origins and varieties) underwent during a long-term frozen storage. Firstly, the volatiles responsible for the pleasant aroma of virgin olive oil will be considered, and afterwards, the oxidation markers will be studied in-depth.

### 3.2. Green/Fruity-Related Volatile Compounds of the Long-Term Frozen Samples

The volatile compounds responsible for the pleasant aroma of virgin olive oil decreased throughout the storage in the CVOO sample, in contrast to the FVOO sample where they remained stable. However, in the case of the long-term-frozen olive oil samples, significant (*p* < 0.05) differences were not observed between samples stored for one year (first batch) or ten years (third batch), except for *(Z)*-3-hexenyl acetate, although a downward trend was observed for most compounds (Table 4). These results are in line with those observed in the FVOO samples, although these samples were stored for a longer period and included a wider variety of samples. They also agree with those results obtained by [6], who observed that *(E)*-3-hexenal and 1-penten-3-one, among others, maintained their concentration during frozen storage. Likewise, ref. [43] reached the conclusion that storage at low temperatures preserved the concentration values of C6 volatile compounds content in extra-virgin olive oils.

On the other hand, ref. [30] tested various storage temperatures and observed that low temperatures allowed for samples to be preserved for certain period with little variation in their volatile profile. However, they concluded that storage at +4 °C was better than at −20 °C because the oxidation indicators (hexanol and hexanal/*(E)*-2-hexenal ratio) were the lowest.

### 3.3. Study of the Oxidation Markers of the Long-Term-Frozen Samples

As mentioned above, the proposed oxidation markers are four aldehydes (pentanal, hexanal, heptanal, and nonanal) and four acids (acetic, propanoic, butanoic, and hexanoic acids). The aldehydes related to the rancid sensory defect are generated due to the breakdown of hydroperoxides derived from unsaturated fatty acids, which occur from the moment the oil is produced [2,18]. During the storage, CVOO and FVOO samples showed significant (*p* < 0.05) increases in the concentrations of the chosen markers related to the oxidative deterioration of virgin olive oil [7]. In fact, these results are in concordance with those obtained in the 32 long-term-frozen samples, where there was a significant increase in the concentrations of these aldehydes between the first and third batches. Pentanal presented 73.6% higher concentration in the third batch than in the first batch, while in the case of nonanal, the increase was of 54.4%. Thus, the concentration of both aldehydes was higher over time but reached a lower concentration than those previously reported at ambient temperature storage (20 °C) [17,37].

**Figure 2:**
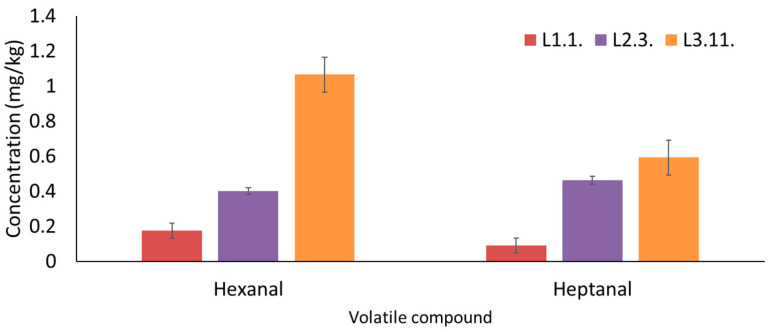
Variation in hexanal and heptanal concentrations (mg/kg) between the selected samples of the three batches; the different letters above each bar indicate significant differences (*p* < 0.05). The samples employed were sample 1 from the first batch, sample 3 from the second batch, and sample 11 from the third batch.

The other oxidation-marker aldehyde was hexanal, which is formed from 13-hydroperoxide of the linoleic acid via the LOX pathway during the oil extraction process and via lipid autooxidation during storage [2,18]. Figure 2 shows the concentration of hexanal in a sample from each batch, and it is observed that it follows an upward trend. The results showed that the hexanal concentration was 83.5% higher in the third-batch samples than in the first-batch samples due to the longer storage. Caipo et al. [38] conducted a study on the effect of storage under different conditions on EVOO composition and obtained an increase of 4% and 10.2% after twelve months of freezing and room temperature storage, respectively. As in the FVOO, and in contrast to the CVOO sample, the hexanal concentration increased because the samples were not oxidized rapidly to hexanoic acid. The fourth aldehydic marker, entirely generated by oxidation, was heptanal, even though it has not been considered as a usual oxidation marker for oils stored in dark conditions [21]. Heptanal showed significant (*p* < 0.05) differences, such as its concentration being 62.5% higher in the third batch of samples than in the first batch (Figure 2). A comparison of the average concentration was performed, and an increment was observed from the first to the third batch of 67.3% for hexanal and of 40.6% for heptanal.

The behavior of the aldehydes in the long-term storage was the same as in the FVOO sample. Thus, the aldehydic markers are suitable for measuring the oxidative process, highlighting that the oxidation still occurs even though the samples had been stored at low temperature and is similar independently of the variety or origin. Storage at −20 °C preserves the aldehydes related to the green/fruity sensory attributes, whereas those derived from oxidation process increase their concentration along the storage but at a lower rate. This is in contrast to the CVOO sample, where the LOX-derived aldehydes were displaced by the oxidation markers aldehydes, whose concentrations increased faster than in the freezing-storage samples.

The other compounds used as oxidation markers were carboxylic acids. The acids come from the oxidation of aldehydes as a consequence of oil oxidation during storage [7,38]. Table 5 shows the concentrations of the acid markers (acetic, propanoic, butanoic, and hexanoic acids) observed in different samples of each batch.

The concentrations of acids varied significantly (*p* < 0.05) between batches (Table 5). Acetic acid showed the greatest difference between the three groups, with an average difference of 50% between the first and second batch and of 45% between the second and third batch. The results obtained for the 32 long-term-frozen samples agree with those published by [38]. However, the concentrations of propanoic and butanoic acids showed the least differences, but significant (*p* < 0.05), the smallest difference being observed between samples of the first and third batch of 66.7% for propanoic acid and 50% for butanoic acid. In contrast, other authors did not observe the same variation for propanoic acid, which may be due to the time and conditions of storage being different [17,21]. Additionally, the hexanoic acid concentration showed significant differences (*p* < 0.05) between the different batches.

Therefore, the aldehydic and acid markers showed the same behavior in the 32 long-term-frozen samples than in the FVOO sample. These compounds were able to show the oxidative degradation of the samples, showing that those with longer storage had higher concentrations of these compounds. However, the generation of the eight markers was at a lower rate than for the CVOO samples.

### 3.4. Multivariate Statistical Analysis of the Long-Term-Frozen Samples

**Figure 3:**
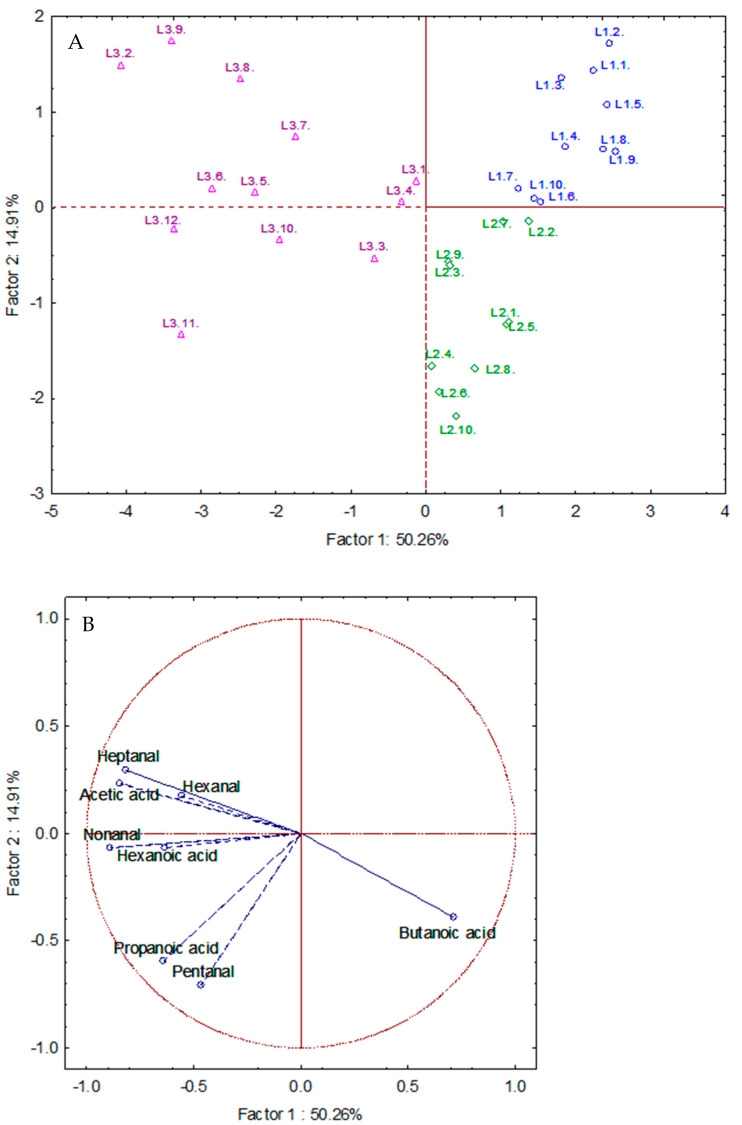
(**A**) PCA of the first (blue), second (green), and third (purple). (**B**) Scores of the variables applied.

A principal component analysis (PCA) was performed on the 32 long-term-frozen samples, considering as variables the selected oxidation markers. Figure 3 shows the results of the PCA in a standardized plot, which allows for presenting the different samples sorted by groups in a two-dimensional projection. It can be observed that the different groups of samples are separated in different quadrants due to their chemical differences. Factor 1 separates the first and second batch from the third one, whereas Factor 2 separates the first and second batch. In addition, the third batch shows a greater spread, while the first and second batches are less dispersed. However, the second and third batches are increasingly influenced by the oxidation markers. As can be seen in Figure 3, no variable is in the first quadrant where first-batch samples are, whereas all variables, except butanoic acid, are in the negative part of X axis, where the third-batch samples are.

These results indicate that freezing preserves the samples from oxidation, delaying the production of aldehydes and acids, but the period during which samples remain unaltered under freezing is limited, being between 1 and 6 years. In comparison with the room-temperature storage (usual procedure), this degradation process occurs much slower under freezing, preserving virgin olive oil quality longer.

### 3.5. Sensory Analysis

The sensory evaluation of the samples was carried out to determine the appearance and evolution of rancidity. The rancid sensory defect can have different descriptors associated, which were considered when studying the results.

**Figure 4:**
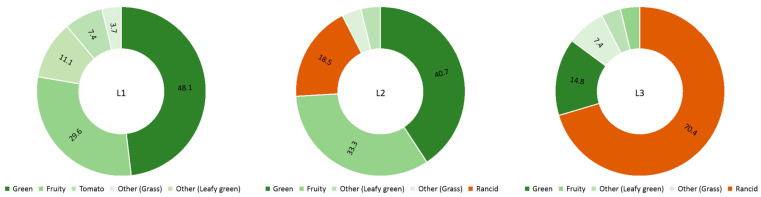
Average sensory profile of the first (L1), second (L2), and third (L3) batch. Attributes are expressed as % of assessors perceiving them. The green and orange palette correspond to green/fruity and rancid attributes, respectively.

As well as for the FVOO and the information reported by other authors [6,31], in the case of the first batch samples (frozen only for a year), the rancid sensory defect was not detected by the assessors (Figure 4). The samples of the second batch (L2) showed different intensities for the rancid defect, ranging from 0.02 to 0.48. These samples belonged to the 2016/17 season, so they had been frozen for 6 years. The intensity of the rancid sensory defect was much lower than the obtained for the CVOO sample after 4 months of storage, demonstrating the protective effect of freezing on the appearance of rancidity. Samples L2.9. and L2.10. had very-low-intensity values, thus showing that the appearance of the rancid sensory defect was incipient and indicating that the oil was no longer an extra-virgin olive oil. In fact, the description of the defect made by the assessors in these samples was as a slight rancid smell. The rest of the samples in this group presented different intensities of the defect being described as slightly rancid. In all the samples of the second batch, the rancid was underneath the fruity-green attributes (Figure 4), remarking what was observed in the FVOO and reported by other authors [32,43] who found that during frozen storage, LOX-derivate volatile compounds do not seem to be removed. These results indicated that the samples started to show secondary volatile-oxidation compounds and that they were perceived by the assessors, which agrees with the information obtained from the volatile compounds.

The samples of the third batch showed a higher intensity of the rancid sensory defect (Figure 4), which was described by all assessors with the term rancid itself or with related descriptors such as paint, fishy, or oily. This indicates that these samples already showed rancidity although at relatively low levels, the intensity of all L3 samples being lower than in the CVOO sample after 6 months of storage. Samples from the third batch had been frozen for 10 years and therefore confirmed that while freezing slows the appearance of the defect and the oxidative process, it eventually occurs. This deterioration of extra-virgin olive oil is caused by auto-oxidation (due to the absence of light and the low temperature applied) of the frozen oil, which means that it always occurs but at a much slower rate than under ambient conditions.

The information of the volatile compounds explained the results obtained by the sensory analysis. Although, in some cases, such as for the L3.6. or L3.11. samples, the rancid sensory defect continued to be masked by the initial volatiles of the oil, which mainly contribute to the green and fruity aroma, such as in L2 samples. The oxidative process could vary due to the large variability of the samples, affecting the final perception of the sensory attributes.

Thus, this work shed light on samples with a great variability that undergo different storage conditions. Despite the large number of varieties and production areas, common volatile compounds are generated during the oxidative process of virgin olive oil. The oxidation of unsaturated fatty acids leads to the formation of aldehydes, and when it progresses, aldehydes are oxidized to acids. Thus, to follow the oxidation process, several volatile markers, such as pentanal, hexanal, heptanal, nonanal, acetic acid, propanoic acid, butanoic acid, and hexanoic acid, are proposed. These markers were chosen based on the results obtained from CVOO and FVOO samples.

### 3.6. Conclusions

The characterization of 32 long-term-frozen virgin olive oil samples revealed that freezing preserves the volatile profile and sensory properties for up to one year, similar to fresh samples. However, after six years, incipient rancidity appeared due to increased oxidation compounds. The third batch showed the highest rancid sensory defect and were related to the presence of aldehydes and acids. The eight proposed markers made it possible to assess the level of oxidation and a systematically compare the rancidity level of the samples, also explaining the sensory perception.

Future trends and practical applications from these findings indicate that freezing can be an effective method for preserving virgin olive oil (VOO) quality over extended periods, when this period is needed for different reasons. While freezing slows oxidation and delays rancidity, the preservation benefits are limited to a timeframe of 1 to 6 years, depending on storage conditions. All this without taking into account a very important aspect to be evaluated in future studies, which is the effect of freezing on the phenolic compounds in olive oil. The loss of phenols during freezing would mean that once the oil is thawed, and its oxidative stability would be much lower than it was at the beginning. This study also suggests that monitoring specific volatile compounds—aldehydes and acids—serves as a reliable marker of oxidation, facilitating the prediction and assessment of VOO freshness over time. These markers allow for a more systematic quality control process in frozen storage contexts, potentially aiding producers in making informed decisions regarding inventory and product labeling.

## Figures and Tables

**Table 1:** Long-term frozen samples code, harvesting season, variety, and geographical origin of the samples.

Sample Code	Harvesting	Variety	Origin	Sample Code	Harvesting	Variety	Origin
L1.1.	2021/22	Picual	Spain	L2.7.	2016/17	Arbequina	Italy
L1.2.	2021/22	Arbequina-Picual	Spain	L2.8.	2016/17	Manzanilla, Hojiblanca, Picual	Spain
L1.3.	2021/22	Picual	Spain	L2.9.	2016/17	-	Spain
L1.4.	2021/22	Arbequina	Spain	L2.10.	2016/17	Koroneiki	Greece
L1.5.	2021/22	Picuda, Picual, Hojiblanca	Spain	L3.1.	2012/13	Hojiblanca	Spain
L1.6.	2021/22	Picuda	Spain	L3.2.	2012/13	Hojiblanca	Spain
L1.7.	2021/22	Hojiblanca	Spain	L3.3.	2012/13	Hojiblanca	Spain
L1.8.	2021/22	Cornicabra	Spain	L3.4.	2012/13	Hojiblanca	Spain
L1.9.	2021/22	Picual	Spain	L3.5.	2012/13	Hojiblanca	Spain
L1.10.	2021/22	Amarga, Picual	Spain	L3.6.	2012/13	Hojiblanca	Spain
L2.1.	2016/17	Biancolilla	Italy	L3.7.	2012/13	Hojiblanca	Spain
L2.2.	2016/17	Arbequina	Morocco	L3.8.	2012/13	Hojiblanca	Spain
L2.3.	2016/17	Manzanilla	Spain	L3.9.	2012/13	Hojiblanca	Spain
L2.4.	2016/17	Leccino, Pendolino	Croatia	L3.10.	2012/13	Hojiblanca	Spain
L2.5.	2016/17	Picholine, Leccio del Corno	Croatia	L3.11.	2012/13	Hojiblanca	Spain
L2.6.	2016/17	Frantoio	Italy	L3.12.	2012/13	Hojiblanca	Spain

**Table 2:** Physicochemical parameters (acidity index, peroxide value, K_232_, K_270_) ± SD of the FVOO and CVOO samples during the storage.

Sample	Acidity Index (% m/m Expressed as Oleic Acid)	Peroxide Value (meq O_2_/kg Oil)	K_232_	K_270_
Control sample (VOO-0)	0.45 ± 0.01	4.00 ± 0.06	1.50 ± 0.04	0.19 ± 0.01
CVOO-1	0.48 ± 0.02 **	4.20 ± 0.08 **	1.58 ± 0.04 **	0.21 ± 0.01 **
CVOO-2	0.50 ± 0.02 **	5.73 ± 0.07 **	1.65 ± 0.03 **	0.22 ± 0.01 **
CVOO-3	0.65 ± 0.02 *	5.71 ± 0.10 **	1.67 ± 0.04 **	0.24 ± 0.01 *
CVOO-4	0.70 ± 0.03 *	5.75 ± 0.09 **	1.68 ± 0.03 **	0.25 ± 0.01 *
CVOO-5	0.73 ± 0.03 *	6.72 ± 0.11 **	1.69 ± 0.02 *	0.25 ± 0.01 *
CVOO-6	0.81 ± 0.01 *	7.41 ± 0.12 *	1.73 ± 0.03 *	0.26 ± 0.01 *
FVOO-1	0.45 ± 0.02 **	4.14 ± 0.09 **	1.55 ± 0.05 **	0.19 ± 0.01 **
FVOO-2	0.46 ± 0.02 **	5.67 ± 0.08 **	1.59 ± 0.04 **	0.19 ± 0.02 **
FVOO-3	0.48 ± 0.02 *	5.67 ± 0.08 **	1.61 ± 0.06 **	0.19 ± 0.01 *
FVOO-4	0.51 ± 0.02 *	5.71 ± 0.07 **	1.62 ± 0.04 **	0.20 ± 0.01 *
FVOO-5	0.52 ± 0.01 *	6.55 ± 0.10 **	1.62 ± 0.02 *	0.20 ± 0.01 *
FVOO-6	0.55 ± 0.02 *	6.74 ± 0.10 *	1.64 ± 0.03 *	0.20 ± 0.01 *

Note: ** expressed non-significant differences for this parameter between commercially stored virgin olive oil (CVOO) and frozen-stored virgin olive oil (FVOO) samples after the same storage time (*p* < 0.05); * expressed significant differences for this parameter between CVOO and FVOO samples after the same storage time (*p* < 0.05).

**Table 3:** Intensity means of the main attributes perceived in the control sample by the assessors.

Sample	Fruity	Green	Tomato	Other (Apple)	Other (Sweet)	Rancid
VOO-0	2.00	2.50	1.50	1.00	1.00	n.d.
CVOO-1	1.50	2.00	1.00	0.50	0.50	n.d.
CVOO-2	1.00	1.00	0.25	0.25	n.d.	n.d.
CVOO-3	1.00	0.50	n.d.	n.d.	n.d.	0.25
CVOO-4	0.50	1.00	n.d.	n.d.	n.d.	0.50
CVOO-5	0.50	0.50	n.d.	n.d.	n.d.	2.00
CVOO-6	0.00	0.00	n.d.	n.d.	n.d.	3.00

Note: n.d., not detected.

**Table 4:** Concentration ± SD ^1^ (mg/kg) of green/fruity-related volatile compounds.

Sample	*(Z)*-3-Hexenyl Acetate	Hexanol	*(Z)*-2-Hexenol	*(E)*-3-Hexenal
L1.3.	0.151 ± 0.001	0.097 ± 0.001	0.021 ± 0.003	0.125 ± 0.001
L1.7.	0.203 ± 0.004	0.159 ± 0.003	0.039 ± 0.001	0.144 ± 0.001
L1.10.	0.176 ± 0.012	0.086 ± 0.010	0.057 ± 0.004	0.132 ± 0.001
L2.5.	0.066 ± 0.002	0.165 ± 0.007	0.022 ± 0.006	0.032 ± 0.001
L2.8.	0.101 ± 0.018	0.251 ± 0.023	0.012 ± 0.001	0.032 ± 0.003
L2.9.	0.148 ± 0.010	0.119 ± 0.001	0.086 ± 0.006	0.045 ± 0.003
L3.7.	0.094 ± 0.006	0.119 ± 0.008	0.013 ± 0.000	0.018 ± 0.002
L3.8.	0.044 ± 0.003	0.183 ± 0.008	0.023 ± 0.002	0.019 ± 0.001
L3.9.	0.074 ± 0.004	0.122 ± 0.002	0.040 ± 0.009	0.044 ± 0.001

Note: ^1^, standard deviation; the analyses were performed in triplicate (n = 3).

**Table 5:** Concentration ± SD ^1^ (mg/kg) of acids in samples of the three batches.

Sample	Acetic Acid	Propanoic Acid	Butanoic Acid	Hexanoic Acid
L1.2.	0.162 ± 0.015	0.003 ± 0.001	0.008 ± 0.002	0.001 ± 0.001
L1.8.	0.237 ± 0.060	0.008 ± 0.001	0.008 ± 0.001	0.001 ± 0.000
L1.9.	0.244 ± 0.050	0.007 ± 0.002	0.007 ± 0.001	0.001 ± 0.000
L2.1.	0.459 ± 0.019	0.013 ± 0.001	0.009 ± 0.002	0.004 ± 0.001
L2.4.	0.529 ± 0.011	0.013 ± 0.001	0.010 ± 0.001	0.004 ± 0.001
L2.6.	0.761 ± 0.200	0.017 ± 0.003	0.009 ± 0.001	0.007 ± 0.002
L3.2.	4.588 ± 0.086	0.024 ± 0.001	0.016 ± 0.003	0.017 ± 0.001
L3.6.	4.661 ± 0.223	0.024 ± 0.002	0.018 ± 0.003	0.014 ± 0.001
L.3.12.	3.223 ± 0.034	0.025 ± 0.001	0.019 ± 0.004	0.019 ± 0.000

Note: ^1^, standard deviation.

## Data Availability

The original contributions presented in the study are included in the article/Supplementary Material, further inquiries can be directed to the corresponding author.

## Supplementary Materials

The following supporting information can be downloaded at: https://www.mdpi.com/article/10.3390/foods13233764/s1, Figure S1: Evaluation sheet for the quantitative-descriptive analysis (QDA).
