# Supplementary material for: Impact of Frozen Storage on Sensory, Physicochemical, and Volatile Compounds Parameters of Different Extra Virgin Olive Oils
Source: Foods. 2024 Nov 24;13(23):3764. doi: 10.3390/foods13233764 (PMC11640403; doi:10.3390/foods13233764)
Supplement: Supplementary file 1 [file foods-13-03764-s001.zip › foods-3227503-supplementary.pdf]

Supplementary Materials

EVALUATION SHEET

Assessor Name:

Sample Code:

Date:

Positive attributes

- Green ____________________
- Fruity ____________________
- Tomato ____________________
- Other: ____________________
- Other: ____________________

Comments:

Sensory defects

- Rancid ____________________
- Fusty/Muddy sediment ____________________
- Greasy ____________________
- Winey/Vinegary ____________________
- Musty/Humid ____________________
- Other: ____________________
- Other: ____________________

Comments:

Figure S1. Evaluation sheet for the quantitative-descriptive analysis (QDA).
